# Low Dose Gamma Irradiation Does Not Affect the Quality or Total Ascorbic Acid Concentration of “Sweetheart” Passionfruit (*Passiflora edulis*)

**DOI:** 10.3390/foods4030376

**Published:** 2015-08-26

**Authors:** John B. Golding, Barbara L. Blades, Shashirekha Satyan, Lorraine J. Spohr, Anne Harris, Andrew J. Jessup, John R. Archer, Justin B. Davies, Connie Banos

**Affiliations:** 1 New South Wales Department of Primary Industries, Ourimbah NSW 2258, Australia; E-Mails: barbara.blades@dpi.nsw.gov.au (B.L.B.); shashirekha.satyan@dpi.nsw.gov.au (S.S.); lorraine.spohr@dpi.nsw.gov.au (L.J.S.); anne.harris@dpi.nsw.gov.au (A.H.); andrew.jessup@dpi.nsw.gov.au (A.J.J.); john.archer@dpi.nsw.gov.au (J.R.A.); 2 School of Environmental and Life Sciences, University of Newcastle, Ourimbah NSW 2258, Australia; 3 Australian Nuclear Science and Technology Organisation, Lucas Heights NSW 2234, Australia; E-Mails: jbd@ansto.gov.au (J.B.D.); cbx@ansto.gov.au (C.B.)

**Keywords:** low dose irradiation, quality, nutrition, storage, phytosanitary, vitamin C

## Abstract

Passionfruit (*Passiflora edulis*, Sims, cultivar “Sweetheart”) were subject to gamma irradiation at levels suitable for phytosanitary purposes (0, 150, 400 and 1000 Gy) then stored at 8 °C and assessed for fruit quality and total ascorbic acid concentration after one and fourteen days. Irradiation at any dose (≤1000 Gy) did not affect passionfruit quality (overall fruit quality, colour, firmness, fruit shrivel, stem condition, weight loss, total soluble solids level (TSS), titratable acidity (TA) level, TSS/TA ratio, juice pH and rot development), nor the total ascorbic acid concentration. The length of time in storage affected some fruit quality parameters and total ascorbic acid concentration, with longer storage periods resulting in lower quality fruit and lower total ascorbic acid concentration, irrespective of irradiation. There was no interaction between irradiation treatment and storage time, indicating that irradiation did not influence the effect of storage on passionfruit quality. The results showed that the application of 150, 400 and 1000 Gy gamma irradiation to “Sweetheart” purple passionfruit did not produce any deleterious effects on fruit quality or total ascorbic acid concentration during cold storage, thus supporting the use of low dose irradiation as a phytosanitary treatment against quarantine pests in purple passionfruit.

## 1. Introduction

Passionfruit are hosts to a range of quarantine pests including Queensland fruit fly, *Bactrocera tryoni* (Froggatt). An effective postharvest disinfestation treatment is essential if the fruit is to be traded in certain domestic and export markets. Irradiation is a technologically proven, viable and scientifically sound disinfestation treatment [1]. Moreover, irradiation is increasingly becoming an approved and agreed application in the world trade of food and horticultural products [1]. Generic irradiation treatments have been approved by U.S. Department of Agriculture—Animal and Plant Health Inspection Service (USDA-APHIS) at doses of 150 Gy for tephritid fruit flies and 400 Gy for all insects except pupal and adult Lepidoptera [2]. Further APHIS rulings and new rulings by the International Plant Protection Convention have approved minimum doses for six fruit fly pests and 14 other plant insect pests regardless of the host product, at doses ranging from 60 to 300 Gy [2]. A maximum of 1000 Gy is approved for use on fresh fruits and vegetables [3], and therefore doses up to this level can be used for phytosanitary purposes. Based on these recommendations, irradiation doses of 0, 150, 400 and 1000 Gy were selected for this study.

There has been little published research on the effects of irradiation as a quarantine treatment on passionfruit quality. Kader reported that passionfruit are moderately tolerant to ≤1000 Gy irradiation, but provided no data [4]. Another study reported on the effects of low dose irradiation on yellow passionfruit respiration rates [5], but the effects of irradiation on fruit quality and acceptability were not reported.

Vitamin C is an essential water-soluble vitamin with important human health benefits [6]. Vitamin C is defined as the generic term for all compounds exhibiting the biological activity of L-ascorbic acid (AA). AA is the principal biologically active form but l-dehydroascorbic acid (DHAA), an oxidation product, also exhibits biological activity [7]. Total ascorbic acid levels (AA + DHAA) in fruits and vegetables can be influenced by various factors such as genotypic differences, preharvest climatic conditions and cultural practices, maturity and harvesting methods, and postharvest handling procedures, including irradiation [8]. AA is inherently unstable in solution and is one of the most sensitive vitamins to irradiation, where the effects of irradiation are influenced by exposure to oxygen, storage and temperature, as well as the pH of the food matrix or storage medium [9,10]. Irradiation has been reported to result in some AA being converted to DHAA [9] and since DHAA can also be easily converted into AA in the human body it is important to measure both AA and DHAA in fruits and vegetables and report total ascorbic acid activity [8].

There have been no reported studies of low dose irradiation on the quality and total ascorbic acid concentration of the purple “Sweetheart” passionfruit, one of the main purple passionfruit cultivars grown in Australia. This experiment examined the effect of low dose irradiation (0, 150, 400 and 1000 Gy) treatment on “Sweetheart” purple passionfruit quality (overall fruit quality, colour, weight loss, fruit firmness, TSS, TA) and total ascorbic acid concentration (AA plus DHAA) after storage at 8 °C.

## 2. Experimental Section

### 2.1. Source of Fruit

Purple passionfruit (*Passiflora edulis*, cultivar “Sweetheart”) were sourced from a commercial farm in Woombye, Queensland, Australia in April 2014. Fruit were harvested at commercial maturity standard and directly sent to the Postharvest Laboratory at NSW Department of Primary Industries (DPI) at Ourimbah NSW.

At NSW DPI, the fruit were randomly allocated to one of 48 groups of 10 fruit. Each group of 10 passionfruit was placed into a netted bag. Before irradiation, bags of fruit were randomly selected and labelled to correspond to a replicate (1, 2 or 3), treatment (0, 150, 400 or 1000 Gy) and assessment time (1 or 14 days). The fruit were then stored in cardboard cartons at 8 °C.

### 2.2. Experimental Design

The experiment was designed as a split plot with three replicates. The four irradiation treatments (0, 150, 400 and 1000 Gy) were assigned to whole plots (total of 12) while the two assessments were assigned to sub-plots giving a total of 24 experimental units. Each experimental unit consisted of two bags of ten passionfruit; one bag was assessed for fruit quality at NSW DPI, whilst one bag was sent under refrigeration for total ascorbic acid (AA plus DHA) analysis at the National Measurement Institute in Melbourne.

### 2.3. Gamma Irradiation

The gamma irradiation treatment was conducted at the Gamma Technology Research Irradiator facility at Australian Nuclear Science and Technology Organisation, Lucas Heights, NSW, using a cobalt-60 plaque source. During treatment the boxes were positioned on a stainless steel rig parallel to the plaque source. The 0 Gy treatments were the untreated controls. These control fruit were not irradiated, but were held at a similar holding temperature and for a similar period of time to the fruit which were undergoing irradiation treatment. Irradiation treatments were randomised within each replication and doses were delivered independently. For example, Replicate 1/150 Gy was treated at a separate time to both Replicate 2/150 Gy and Replicate 3/150 Gy.

To record the radiation exposure and to ensure the correct dosage, dosimeters were positioned throughout the array of bags of fruit in the cardboard boxes at the expected minimum and maximum dose zones, taking into consideration locations of non-homogeneous product distribution. Fricke dosimeters [11] were positioned at the front and in between the bags. Additional dosimeters were attached to the outside of one box to provide a reference to the minimum and maximum doses (the monitoring position). The dosimeters were calibrated for readings 50–350 Gy and the 150, 400 and 1000 Gy bags from Replicate 1 were used to carry out a dose mapping exercise at approximately 150 Gy intervals to 450 Gy. The locations of minimum and maximum doses were found and dose mapping repeated twice with dosimeters at those locations. This dose mapping information was used to process the remaining bags of fruit at their target doses. The dose rate was approximately 9.2 Gy min^−1^. Using the dosimeter readings the minimum, maximum and average dose for each replicate/irradiation level were calculated. After treatment, the fruit were stored at 8 °C in cardboard cartons, where the relative humidity within the cartons was 92%–97%.

### 2.4. Storage and Assessment Times

After irradiation treatment, fruit were returned to NSW DPI Ourimbah and stored at 8 °C for one or two days (assessment 1) or 14 or 15 days (assessment 2). For the first fruit quality assessment, non-destructive observations were made one day after the irradiation treatment with the destructive testing (total ascorbic acid, TSS and TA analysis) conducted two days after the irradiation treatment. The final fruit quality assessment (assessment 2) was made 14 days after treatment for non-destructive observations and 15 days after irradiation for destructive testing (total ascorbic acid, TSS and TA analysis).

### 2.5. Fruit Quality Assessments

The fruit was removed from 8 °C storage and allowed to reach 20 °C before any quality assessments were conducted. To prevent any bias in sampling, the order of sample assessment was randomly allocated and ten fruit in each bag were assessed.

#### 2.5.1. Subjective Fruit Quality Assessments

Subjective fruit quality was evaluated independently by three trained assessors.

Overall fruit quality was assessed using a subjective four point scoring system; 1 = high quality fruit with glossy skin and no of signs of dehydration, shrivelling, decay or bruises; 2 = acceptable fruit quality with dull skin and slight signs of shrivelling, bruises and softness; 3 = unacceptable fruit with dull skin and moderate signs of shrivelling, browning, dryness, bruises and softness; and 4 = poor quality fruit with evident signs of shrivelling, pitting, significant softness, decay.

Fruit shrivel was scored using a five point scoring system; 1 = no skin shrivel, full plump fruit; 2 = slight shrivel (<10% shrivel); 3 = light shrivel (<25% shrivel); 4 = moderate shrivel (50% shrivel); and 5 = severe deep shrivel.

Fruit firmness was scored using a four point scoring system; 1 = hard; 2 = slightly soft; 3 = soft; 4 = very soft and unacceptable.

Stem condition was appraised visually and scored according to the following four point scoring system; 1 = green; 2 = predominantly yellow with some green; 3 = all yellow; 4 = brown and withered.

Each fruit was inspected for rot development and scored according to the following four point scale: 1 = no rot; 2 = start of a rot; 3 = obvious rot (<1 cm^2^); 4 = significant rot (>1 cm^2^).

Off odours were assessed after cutting the fruit open and subjectively assessing it to determine if any “off odours” were present.

#### 2.5.2. Objective Fruit Quality Assessments

Skin colour was quantified using a Minolta Chroma Meter CR400 with a 10mm aperture (Minolta Co. Ltd, Osaka, Japan). The colour parameters “L” (lightness), “a” (redness to greenness), “b” (yellowness to blueness) and hue angle (hue angle = tan^−1^ [“b”/“a”]) were recorded.

The fruit weight of each bag was determined before and after storage using a Kern PLS—2100-2 electronic balance and the percentage weight loss of the fruit during storage was calculated using the following formula:

$$percentage\;weight\;loss=\;\frac{\left(pre\;-\;treatment\;weight\;-\;post\;-\;treatment\;weight\right)\;x\;100}{\left(pre\;-\;treatment\;weight\right)}$$

Each fruit was cut open, the pulp removed and the weights of the pulp, rinds and juice content were determined. The percentage weight of fruit being rind, pulp, juice as well as the pulp/rind ratio were calculated. The juice collected in this way was immediately used to measure total soluble solids (TSS), pH and total acidity (TA).

An Atago PR-32 Palette refractometer was used to determine the TSS, expressed as % Brix. TSS was determined using two aliquots per sample (assessment unit).

The pH and TA of the fresh juice samples were determined using a Mettler Toledo Titration Excellence T50 Titrator with a DG101-SC electrode. Juice aliquots (2 mL) were titrated against 0.1 N NaOH to an end-point of pH 8.2. The TA was expressed as % citric acid (citric acid g/100 mL). The pH of the juice before titration was recorded. Two aliquots of each sample were assayed.

### 2.6. Determination of Total Ascorbic Acid Concentration

Determination of total ascorbic acid concentration was conducted by NATA (National Association of Testing Authorities, Australia) accredited analytical laboratories at the National Measurement Institute (NMI) in Melbourne, Victoria, Australia. The determination of total ascorbic acid was based on a HPLC method adapted from Brubacher *et al.* [12], where the limit of reporting was 1 mg/100 g. In brief, the samples were processed and assayed immediately on receipt to prevent degradation of the ascorbic acid. The pulp from the ten passionfruit from each experimental unit was pooled to form one sample which was then weighed and blended with a metaphosphoric acid and dithiothreitol solution. The dithiothreitol reduces the dehydro forms to their parent acids and stabilises the reduced state. The sample solutions were then filtered through an appropriate pore size filter to obtain a clean filtrate and analysed by HPLC. Determination of the total ascorbic acid concentration was made against known l-ascorbic acid and d-isoascorbic acid standards.

### 2.7. Statistical Analyses

The effects of irradiation level, storage time and their interaction on fruit quality attributes and total ascorbic acid concentration were tested using analysis of variance (ANOVA) in GenStat [13]. The split plot experimental design was accounted for in the ANOVA structure and a separate analysis was carried out for each variable. For variables where multiple fruit were assessed for each experimental unit, the ANOVA blocking strata structure was carefully specified to ensure the appropriate error degrees of freedom were used to test the treatment effects.

The score data were analysed using ANOVA, treating the average score for each experimental unit as continuous data and assuming normally distributed errors. For each experimental unit, the average score of 10 passionfruit, assessed three times (three assessors), was used as the response variable. The normality of errors, an ANOVA assumption, was confirmed by inspection of the residual diagnostic plots produced in GenStat.

All significance tests were carried out at the *p* = 0.05 probability level. Treatment means were compared using the least significant difference (l.s.d.) procedure.

## 3. Results

### 3.1. Dosimetry

The results of the irradiation dosimetry are presented in Table 1 and show that the doses absorbed were both accurate and within the specifications for each dose.

**Table 1:** Delivered irradiation doses (Gy) ± expanded uncertainties to passionfruit. These results include the expanded uncertainties (k = 2) in the dose mapping undertaken to calculate the minimum and maximum doses. Where incremental doses have been delivered (*i.e.*, 400 and 1000 Gy), the uncertainty in each dose fraction has been propagated to calculate the total uncertainty.

Target Irradiation Dose (Gy)	Minimum Dose (Gy)	Maximum Dose (Gy)	Average Dose (Gy)
Replicate 1			
150	146 ± 7	155 ± 9	150 ± 6
400	384 ± 7	410 ± 5	397 ± 3
1000	964 ± 20	1028 ± 24	996 ± 16
Replicate 2			
150	146 ± 7	155 ± 9	151 ± 6
400	378 ± 13	403 ± 16	391 ± 10
1000	952 ± 21	1015 ± 25	984 ± 16
Replicate 3			
150	146 ± 7	155 ± 9	151 ± 6
400	381 ± 13	407 ± 16	394 ± 10
1000	962 ± 26	1026 ± 30	994 ± 20

### 3.2 Fruit Quality

**Figure 1:**
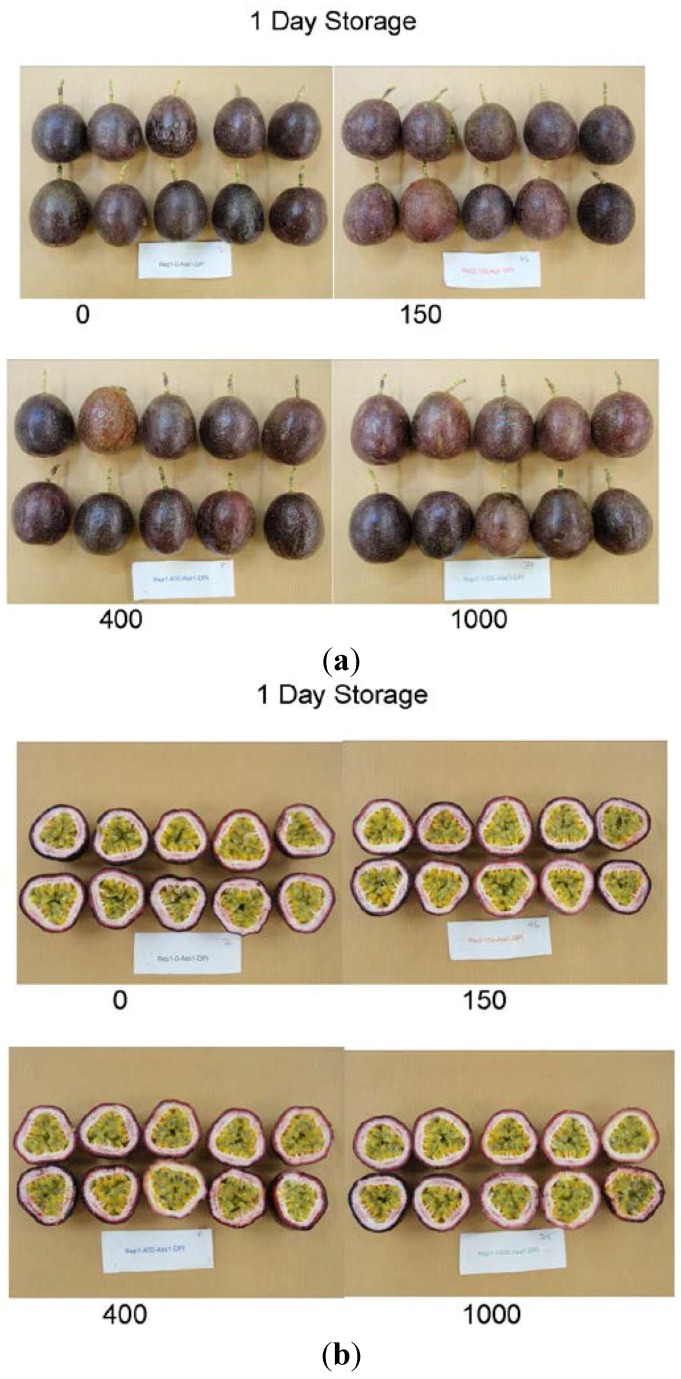
(**a**) Irradiated passionfruit after one day storage at 8 °C. Fruit were treated with 150 Gy (top right), 400 Gy (bottom left) and 1000 Gy (bottom right), or left untreated (0 Gy, top left); (**b**) Irradiated passionfruit after one day storage at 8 °C. Fruit were treated with 150 Gy (top right), 400 Gy (bottom left) and 1000 Gy (bottom right), or left untreated (0 Gy, top left).

**Figure 2:**
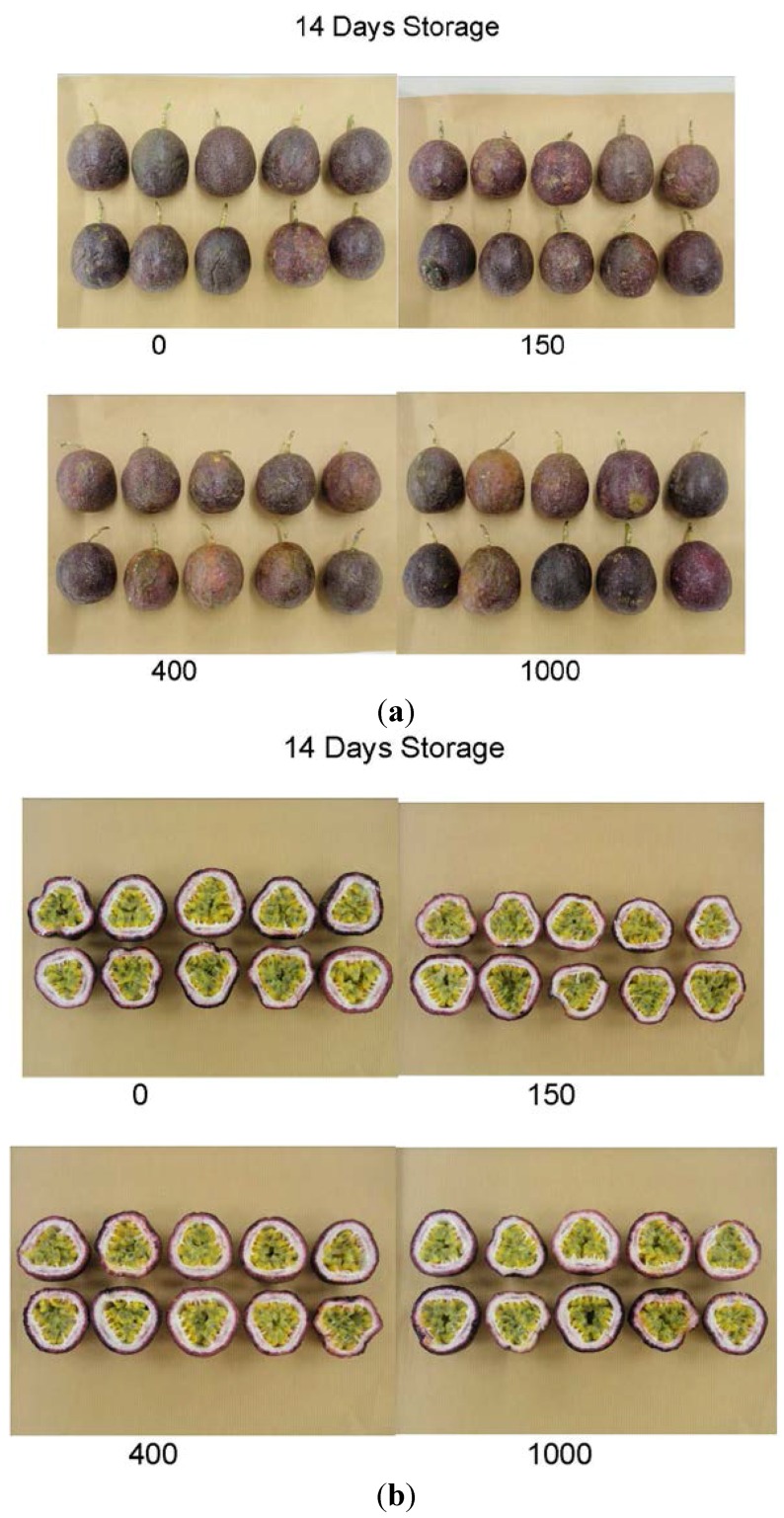
(**a**) Irradiated passionfruit after fourteen days storage at 8 °C. Fruit were treated with 150 Gy (top right), 400 Gy (bottom left) and 1000 Gy (bottom right), or left untreated (0 Gy, top left); (**b**) Irradiated passionfruit after fourteen days storage at 8 °C. Fruit were treated with 150 Gy (top right), 400 Gy (bottom left) and 1000 Gy (bottom right), or left untreated (0 Gy, top left).

After treatment, fruit were stored in air at 8 °C then assessed for quality and total ascorbic acid concentration. Figure 1 and Figure 2 show photographs of the external and internal appearance of representative passionfruit after one day (Figure 1) and fourteen days (Figure 2) storage at 8 °C. A summary of the *p* values for the effects of irradiation dose, storage time and the interaction between irradiation and storage time in passionfruit is presented in Table 2. There was no significant effect of irradiation detected on passionfruit quality (overall fruit quality, skin colour (as determined by Minolta “L”, “a” and “b” values, and hue angle), weight loss, fruit firmness score, pulp recovery, fruit shrivel score, stem score, rots score, TSS, TA, TSS/TA ratio or juice pH). There were no ‘off’ aromas detected in any fruit. Nor was there any detected effect of irradiation on total ascorbic acid concentration. In addition, there was no significant interaction detected between irradiation treatment and storage time in either fruit quality or total ascorbic acid concentration.

**Table 2:** *p* values for the effects of irradiation, storage and the interaction of irradiation and storage treatments on fruit quality attributes and total ascorbic acid concentration. Effects with *p* values <0.05 (in bold) were statistically significant at the 5% level.

Fruit quality attributes	Irradiation (I)	Storage (S)	Interaction (I × S)
Overall fruit quality	0.46	**<0.001**	0.82
Minolta Colour—“L” value	0.96	**0.003**	0.36
Minolta Colour—“a” value	0.73	0.52	0.86
Minolta Colour—“b” value	0.28	0.26	0.36
Minolta Colour—“hue angle” value	0.27	0.73	0.27
Weight loss	0.26	**<0.001**	0.36
Fruit firmness score	0.66	**<0.001**	0.98
Pulp recovery	0.54	0.94	0.47
Fruit shrivel score	0.62	**<0.001**	0.94
Stem score	0.26	**<0.001**	0.48
Rots score	0.22	**<0.001**	0.16
Total soluble solids (TSS)	0.23	0.77	0.23
Titratable acidity (TA)	0.11	0.47	0.65
TSS/TA ratio	0.09	0.37	0.41
Juice pH	0.052	0.53	0.47
Total ascorbic acid concentration	0.21	**<0.001**	0.31

The effects of storage and irradiation treatment on the fruit quality and total ascorbic acid concentration are presented in Table 3 and show that the length of time in cold storage had a significant effect on some fruit quality parameters. For example, overall subjective fruit quality, skin colour (as determined by Minolta “L” value), weight loss %, fruit firmness score, fruit shrivel score, stem score, rots score were all affected by the length of time in storage. Fruit after two weeks storage were of lower overall quality, where these fruit had lost more weight, become softer and started to develop postharvest rots compared to fruit stored for one day.

Time in storage also had a significant effect on the total ascorbic acid concentration in the passionfruit, with a decline from 33.7 mg/100 g after two days in storage to 27.8 mg/100 g after fifteen days in cold storage (Table 3).

**Table 3:** Effect of storage and irradiation treatment (0, 150, 400 and 1000 Gy) on the fruit quality and total ascorbic acid concentration of “Sweetheart” passionfruit stored for one (or two) and fourteen (or fifteen) days at 8 °C. Means with different letters within rows are significantly different (*p* <0.05).

Fruit quality attributes	1 Day Storage					14 Days Storage				
	0 Gy	150 Gy	400 Gy	1000 Gy	1 Day Mean	0 Gy	150 Gy	400 Gy	1000 Gy	14 Days Mean
Overall fruit quality	1.3	1.3	1.4	1.4	**1.4 a**	2.5	2.3	2.7	2.6	**2.5 b**
Minolta Colour—“L” value	30.6	30.4	30.1	30.8	**30.5 a**	31.5	32.4	32.6	31.7	**32.0 b**
Minolta Colour—“a” value	10.4	11.0	10.5	11.2	**10.8 a**	10.3	11.1	11.3	11.5	**11.0 a**
Minolta Colour—“b” value	4.1	4.2	4.6	5.4	**4.6 a**	4.3	5.2	5.2	4.9	**4.9 a**
Minolta Colour—hue angle value	21.8	21.2	24.3	26.5	**23.4 a**	22.8	25.3	25.0	22.6	**23.9 a**
Weight loss (%)	1.05	0.98	1.13	1.11	**1.03 a**	5.45	5.12	5.74	5.86	**5.61 b**
Fruit firmness score	1.4	1.3	1.3	1.4	**1.4 a**	2.4	2.3	2.4	2.5	**2.4 b**
Pulp recovery (%)	39.1	38.9	39.4	41.1	**39.6 a**	38.9	41.3	38.6	39.3	**39.5 a**
Fruit shrivel score	1.6	1.4	1.7	1.5	**1.5 a**	3.0	2.7	2.9	2.8	**2.9 b**
Stem score	1.3	1.3	1.4	1.4	**1.4 a**	2.5	2.8	3.1	3.1	**2.9 b**
Rots score	1.0	1.0	1.0	1.0	**1.0 a**	1.5	1.3	1.7	1.5	**1.5 b**
	**2 Days Storage**					**15 Days Storage**				
Total soluble solids (TSS, Brix%)	16.8	16.3	16.4	16.3	**16.4 a**	16.5	16.5	16.4	16.3	**16.4 a**
Titratable acidity (TA, % citric acid)	2.11	2.33	2.33	2.39	**2.29 a**	2.30	2.36	2.36	2.34	**2.34 a**
TSS/TA ratio	8.0	7.0	7.0	6.8	**7.2 a**	7.2	7.0	7.0	7.0	**7.0 a**
Juice pH	3.72	3.65	3.64	3.66	**3.67 a**	3.69	3.65	3.66	3.72	**3.68 a**
Total ascorbic acid concentration (mg/100 g)	33.3	34.0	35.0	32.3	**33.7 a**	26.7	29.3	27.7	27.3	**27.8 b**

## 4. Discussion

### 4.1. Fruit Quality

Irradiation is becoming an increasingly common postharvest disinfestation tool for use against quarantine pests in horticultural produce [1]. However, information on product tolerance to low dose gamma irradiation and its effects on both fruit nutritional status and quality are limiting its application. This study showed that low dose irradiation did not significantly affect the quality of ‘Sweetheart’ purple passionfruit. Irradiation treatment at all doses (150, 400 and 1000 Gy) did not affect fruit quality (overall fruit quality, skin colour (as determined by Minolta “L”, “a” and “b” values, and hue angle), weight loss, fruit firmness score, pulp recovery, fruit shrivel score, stem score, rots score, TSS, TA, TSS/TA ratio, juice pH or the development of “off” aromas). These parameters are important quality parameters in both purple and yellow passionfruit [14].

There have been few published reports on the effects of low dose irradiation on passionfruit quality. Although not a direct measure of fruit quality, irradiation has been shown to generally delay the respiration of a range of different tropical fruits [15]. However gamma irradiation (250–1000 Gy) has been shown to increase the respiration rate of preclimacteric yellow passionfruit (*Passiflora edulis* Sims f. flavicarpa Degener), and increasing irradiation doses hastened the development of the respiratory climacteric [5]. However no other measures of fruit quality were made [5].

In this study, no interaction was detected between the effects of irradiation treatment and storage time, indicating that the observed storage effect on passionfruit quality was consistent for all irradiation doses. The length of time in cold storage (8 °C) had significant effects on overall subjective fruit quality, skin colour (as determined by Minolta ‘L’ value), weight loss, fruit firmness score, fruit shrivel score, stem score and postharvest rot development. The two week storage at 8 °C assessed in this study is consistent with Australian passionfruit industry marketing conditions; including both transport and marketing.

After two weeks storage, irrespective of irradiation treatment, fruit were of lower overall quality compared to fruit stored for only one day; fruit stored for two weeks had lost more weight, become softer with signs of skin shrivel and started to develop postharvest rots (Figure 1 and Figure 2). These results were expected and have been reported in other purple passionfruit storage studies [16]. For example Shiomi *et al.* showed there was a linear reduction in weight loss with an increase in storage time, with weight loss contributing to fruit softening and shrivel development [17]. The levels of rots increased during the two weeks of storage in this study, and were not affected by any irradiation treatment.

Fruit flavour is primarily a balance between TSS and TA [18]. In this experiment, the levels of TSS, TA and TSS/TA did not significantly change during storage time at 8 °C (Table 3); however other studies have reported a decline in TSS and TA levels during regular storage at 25 °C [16]. The constant level of TSS and TA during storage in this study was probably due to the lower storage temperature (8 °C), as fruit stored at higher temperatures (25 °C) ripen and senesce quicker than fruit stored at lower temperatures [18].

These irradiation and storage results are similar to those in other fruits, such as blueberry and raspberry [19] and contribute to the general observations of low dose gamma irradiation not affecting fruit quality [20,21].

### 4.2. Total Ascorbic Acid Concentration

Total ascorbic acid is an important nutritional parameter of fruits and vegetables. Lee and Kader [8] reviewed many studies which showed that low dose irradiation generally did not affect total ascorbic acid concentration in a range of horticulture produce. For example, low dose gamma irradiation (0, 75, or 300 Gy) had no effect on the levels of total vitamin C and DHAA after treatment and storage of tropical fruits and vegetables, such as capsicums (green and red), lemons, lychees, mandarins (“Ellendale”), mangoes, papaws, and persimmons [22]. Their study also reported that low dose irradiated “Imperial” mandarins and “Kensington Pride” mangoes contained significantly lower total vitamin C content than control fruit [22]. Moy and Wong [23] similarly examined the effect of low dose irradiation (750 Gy) on vitamin C retention in other tropical fruit (star fruit, mango, papaya, rambutan, and lychee) and reported that only star fruit had a significant loss of vitamin C [23]. However when interpreting findings of diminished ascorbic acid, it is important to consider the method of ascorbic acid analysis, both in terms of its reliability and what is actually measured [21]. Total ascorbic acid (AA plus DHAA) is a more reliable indicator of post-irradiation vitamin C [21].

The levels of total ascorbic acid in the passionfruit measured in this study were between 25 and 36 mg/100 g. This is within the upper range of other studies [21,24,25], and may reflect the range of different physiological ages of the fruit, cultivars and analytical methods. In this experiment, total ascorbic acid was determined with a HPLC method adapted from Brubacher *et al.* [12].

The levels of total ascorbic acid concentration in “Sweetheart” passionfruit were not affected by low dose irradiation treatment (Table 3), which is consistent with the finding that effects of phytosanitary doses of irradiation on total ascorbic acid concentration in whole fruits are minimal [21]. A reduction of vitamin C levels with increasing irradiation doses have been reported in passionfruit juice (cited by Dionísio *et al.* [10]). But these observations were made on extracted passionfruit pulp and juice which is a different scenario to the whole living fruit used in the current study.

Handling and storage of horticulture produce significantly affects total ascorbic acid levels [8] and in this study the levels of total ascorbic acid were significantly lower after storage (irrespective of the irradiation treatment). The average concentration of total ascorbic acid after one day storage at 8 °C was 33.7 mg/100 g whilst after 14 days storage at 8 °C, the average total ascorbic acid concentration was 27.8 mg/100 g. This decline in total ascorbic acid following cold storage has been observed in a wide range of horticultural produce [19,20].

## 5. Conclusions

This is the first report on the effects of low dose irradiation on passionfruit quality and total ascorbic acid concentration following treatment and storage. The length of time in cold storage had a much greater influence on fruit quality and total ascorbic acid concentration than any of the low dose irradiation (≤1000 Gy) treatments. These results confirm that low dose irradiation (≤1000 Gy) does not have an impact on fruit quality or total ascorbic acid concentration of “Sweetheart” purple passionfruit, supporting the use of low dose irradiation as a phytosanitary treatment against quarantine pests.
